# Supplementary material for: Novel Driver Strength Index highlights important cancer genes in TCGA PanCanAtlas patients
Source: PeerJ. 2022 Aug 11;10:e13860. doi: 10.7717/peerj.13860 (PMC9375969; doi:10.7717/peerj.13860)
Supplement: Supplemental Information 2 [file peerj-10-13860-s002.zip › COHORTS/genes plots/detailed/2021_8_16_14_9_distribution_events_detailed_females_28.pdf]

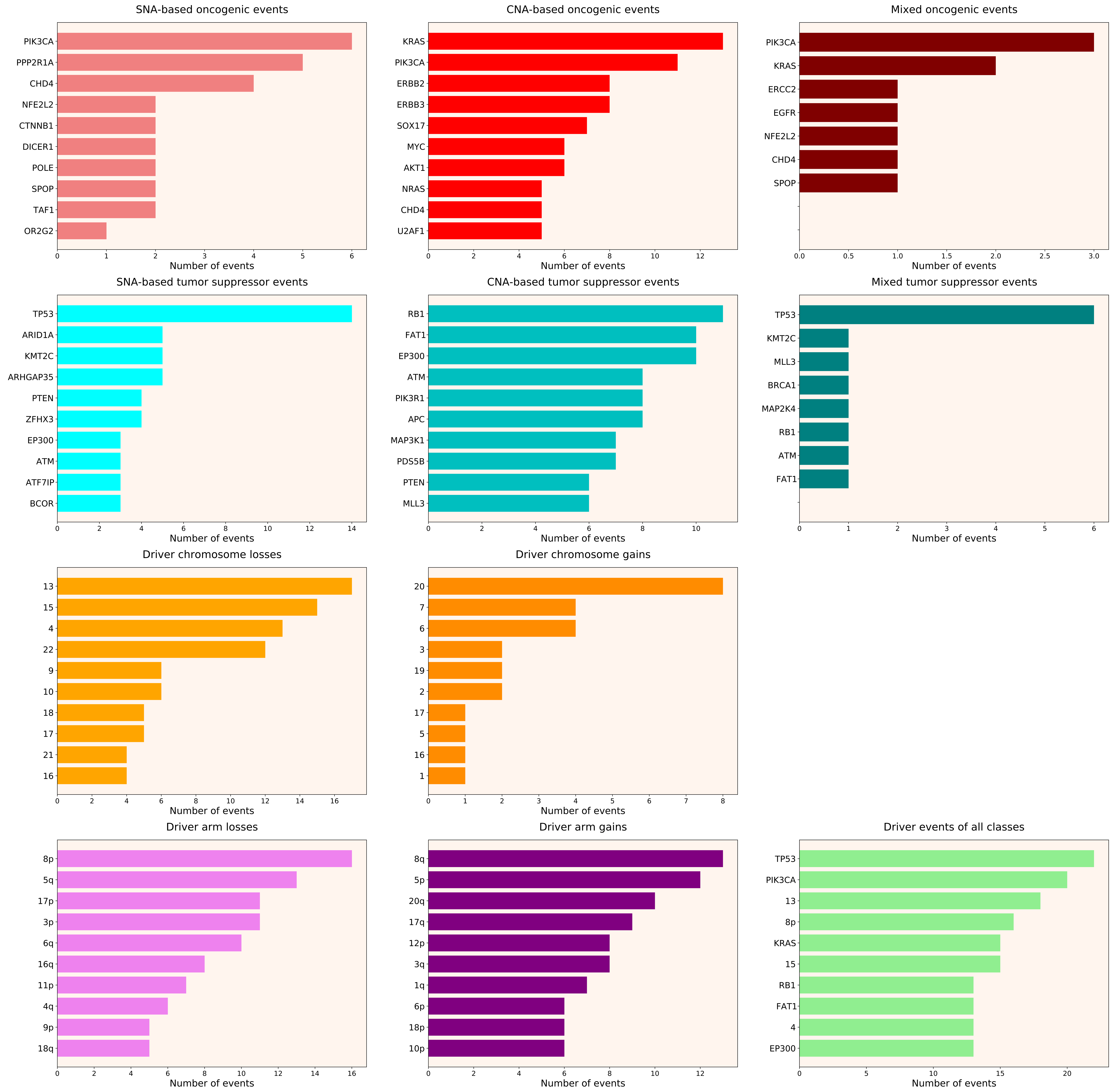
SNA-based oncogenic events
PIK3CA
PPP2R1A
CHD4
NFE2L2
CTNNB1
DICER1
POLE
SPOP
TAF1
OR2G2
Number of events
CNA-based oncogenic events
KRAS
PIK3CA
ERBB2
ERBB3
SOX17
MYC
AKT1
NRAS
CHD4
U2AF1
Number of events
Mixed oncogenic events
PIK3CA
KRAS
ERCC2
EGFR
NFE2L2
CHD4
SPOP
Number of events
SNA-based tumor suppressor events
TP53
ARID1A
KMT2C
ARHGAP35
PTEN
ZFHX3
EP300
ATM
ATF7IP
BCOR
Number of events
CNA-based tumor suppressor events
RB1
FAT1
EP300
ATM
PIK3R1
APC
MAP3K1
PDS5B
PTEN
MLL3
Number of events
Mixed tumor suppressor events
TP53
KMT2C
MLL3
BRCA1
MAP2K4
RB1
ATM
FAT1
Number of events
Driver chromosome losses
13
15
4
22
9
10
18
17
21
16
Number of events
Driver chromosome gains
20
7
6
3
19
2
17
5
16
1
Number of events
Driver arm losses
8p
5q
17p
3p
6q
16q
11p
4q
9p
18q
Number of events
Driver arm gains
8q
5p
20q
17q
12p
3q
1q
6p
18p
10p
Number of events
Driver events of all classes
TP53
PIK3CA
13
8p
KRAS
15
RB1
FAT1
4
EP300
Number of events
